# Functional Analysis of Two Novel *Streptococcus iniae* Virulence Factors Using a Zebrafish Infection Model

**DOI:** 10.3390/microorganisms8091361

**Published:** 2020-09-05

**Authors:** Kar Yan Soh, Jacelyn Mei San Loh, Christopher Hall, Thomas Proft

**Affiliations:** 1 Department of Molecular Medicine & Pathology, School of Medical Sciences, The University of Auckland, Auckland 1142, New Zealand; s.karyan@auckland.ac.nz (K.Y.S.); mj.loh@auckland.ac.nz (J.M.S.L.); 2 Maurice Wilkins Centre for Biomolecular Discoveries, The University of Auckland, Auckland 1142, New Zealand

**Keywords:** nuclease, SpnAi, nucleotidase, S5nAi, *Streptococcus iniae*, virulence, zebrafish infection model, immune evasion

## Abstract

*Streptococcus iniae* is a major fish pathogen that contributes to large annual losses in the aquaculture industry, exceeding US$100 million. It is also reported to cause opportunistic infections in humans. We have recently identified two novel *S. iniae* virulence factors, an extracellular nuclease (SpnAi) and a secreted nucleotidase (S5nAi), and verified their predicted enzymatic activities using recombinant proteins. Here, we report the generation of green fluorescent *S. iniae spnAi* and *s5nAi* deletion mutants and their evaluation in a transgenic zebrafish infection model. Our results show nuclease and nucleotidase activities in *S. iniae* could be attributed to SpnAi and S5nAi, respectively. Consistent with this, larvae infected with the deletion mutants demonstrated enhanced survival and bacterial clearance, compared to those infected with wild-type (WT) *S. iniae*. Deletion of *spnAi* and *s5nAi* resulted in sustained recruitment of neutrophils and macrophages, respectively, to the site of infection. We also show that recombinant SpnAi is able to degrade neutrophil extracellular traps (NETs) isolated from zebrafish kidney tissue. Our results suggest that both enzymes play an important role in *S. iniae* immune evasion and might present potential targets for the development of therapeutic agents or vaccines.

## 1. Introduction

*Streptococcus iniae* is a major fish pathogen originally isolated from the Amazon fresh water dolphin, *Inia geoffrensis* [1]. Since then, a number of fish species have also been reported to be infected by *S. iniae*, including some economically important species, such as tilapia, trout, barramundi and salmon [2]. Globally, these infections contribute to annual losses exceeding US$100 million in the aquaculture industry [3]. *S. iniae* is also an opportunistic human pathogen with most cases reported among elderly Asian populations and among weak or immunocompromised individuals who have a history of handling fish [4,5]. Clinical symptoms of human disease including bacteremic cellulitis are similar to those caused by the important human pathogen, *Streptococcus pyogenes*, which can also subsequently develop into other conditions, such as meningitis, endocarditis, septic arthritis, osteomyelitis and toxic shock [4,6,7]. Following the complete sequencing of the genome of *S. iniae* strain 9117 [8], a number of virulence factors were discovered. Of those that have been documented, many are homologous to those expressed by *S. pyogenes* [5], such as *S. iniae* M-like protein [9,10], hyaluronic acid capsule [11,12,13], C5a peptidase [10] and streptolysin S [14,15].

Analyzing the complete genome of *S. iniae* 9117, we recently identified two *S. iniae* proteins that share some amino acid sequence homology with two important *S. pyogenes* immune evasion factors, *Streptococcus pyogenes* nuclease A (SpnA) [16] and streptococcal 5′-nucleotidase A (S5nA) [17]. These were termed SpnAi and S5nAi, respectively [18]. These *S. iniae* proteins are expressed as precursor proteins with an *N*-terminal signal peptide and a *C*-terminal cell wall anchor domain, including the canonical sortase A recognition motif LPXTG [16,17,18]. Our previous biochemical analysis using a recombinant form of SpnAi (rSpnAi) showed that the enzyme is a calcium and magnesium ion-dependent nuclease that cleaves double stranded linear DNA and chromosomal DNA [18]. Extracellular nucleases produced by *S. pyogenes* (SpnA) [16] and other Gram-positive bacterial species, such as *Staphylococcus aureus* (Nuc) [19], *S. pyogenes* (Sda1) [20] and *S. pneumoniae* (EndA) [21] have been shown to play an important role in immune evasion by degrading the DNA framework of neutrophil extracellular traps (NETs). NETs are released from activated neutrophils and comprise of DNA fibers with antimicrobial proteins such as neutrophil elastase, cathepsin G, and myeloperoxidase. Hence, extracellular nucleases play a crucial role in host immune evasion [22].

Similarly, we have shown that S5nAi is a 5′-nucleotidase that hydrolyzes adenosine monophosphate (AMP), adenosine diphosphate (ADP) and deoxyadenosine monophosphate (dAMP) to produce the immunomodulatory molecules, adenosine (Ado) and deoxyadenosine (dAdo) [18]. Ado is a purine nucleoside that plays important roles in cell signaling. Ado is typically produced under conditions of metabolic stress such as hypoxia and inflammatory tissue damage following an increase in the ATP production [23]. Ado binds specifically to Ado receptors (e.g., A_1_, A_2A_, A_2B_ and A_3_) that are differentially expressed on various innate immune cells, such as neutrophils and macrophages, and have different thresholds for activation [24]. It has been shown that Ado decreases the phagocytic activity of macrophages by suppressing the production of various components required for the macrophage phagocytic action, including proinflammatory cytokines [25], superoxide [26] and nitric oxide [27]. In addition, the binding of Ado to A_2_ and A_3_ receptors on neutrophils inhibit neutrophil degranulation [28].

Several 5′-nucleotidases have also been identified in other Gram-positive bacteria, and have been shown to play an important role in evasion of the host immune response [29]. *Staphylococcus aureus* produces adenosine synthase (AdsA) that hydrolyzes AMP, ADP and ATP to produce Ado [30]. AdsA is also able to convert dAMP into dAdo, which activates caspase-3-mediated apoptosis of macrophages and monocytes [31]. *S. pyogenes* S5nA hydrolyzes AMP and ADP, but not ATP, to generate Ado, and has been shown to increase the survival of the non-virulent bacterium, *Lactococcus lactis*, in whole human blood killing assays [17]. The role of another 5′-nucleotidase, ecto-5′nucleotidase (Nt5e) from *Streptococcus sanguinis* was confirmed in a platelet aggregation assay and an experimental rabbit endocarditis model [32]. Another nucleotidase, *Streptococcus suis* adenosine synthase (Ssads) was reported to impair polymorphonuclear cell-mediated innate immunity [33] and was demonstrated to be an important virulence factor in piglet and mouse infection models [33,34].

Being a major fish pathogen, *S. iniae* has been the most extensively studied streptococcal species in the zebrafish infection model. A major advantage of using the larval zebrafish infection model is that the optical transparency of zebrafish larvae enables visualization of the host immune response to infection in vivo. In zebrafish, innate immunity develops during early stages of development, with fully functional macrophages and neutrophils present as early as 2 days post-fertilization (dpf) [35,36,37]. A morphologically and functionally mature adaptive immune system is not present until 4–6 weeks post-fertilization [38,39,40]. This temporal separation allows the study of the innate immune response independently from the adaptive immune response.

In this study, we show that the deletion of the *spnAi* and *s5nAi* genes in *S. iniae* significantly decreased the nuclease and nucleotidase activity, respectively. We also demonstrated the virulence role of SpnAi and S5nAi in vivo using a *S. iniae*-zebrafish infection model. Our findings suggest that SpnAi and S5nAi contribute to the virulence of *S. iniae* by suppressing innate immune cell function.

## 2. Materials and Methods

### 2.1. Bacterial Strains, Media, Growth Conditions and Electroporation

*S. iniae* strain 9117, a human clinical isolate from a patient with cellulitis (kindly provided by Dr. Sarah Highlander, JVCI, La Jolla, CA), was grown in Todd Hewitt broth medium supplemented with 0.2% yeast extract and 2% proteose peptone (THY+P, BD Biosciences, San Jose, CA, USA) at 37 °C without agitation. *Escherichia coli* DH5α (ATCC^®^ 53868™) was grown in Luria Bertani (LB, BD Biosciences) broth at 37 °C with aeration. Solid THY+P or LB plates were made by adding 1.5% Bacto agar (BD Biosciences) to liquid medium. When appropriate, antibiotics were added to the THY+P or LB medium: spectinomycin at 50 μg/mL for *S. iniae* and 100 μg/mL for *E. coli*; or kanamycin at 200 μg/mL for *S. iniae* and 50 μg/mL for *E. coli*. For all experiments, overnight cultures of *S. iniae* were diluted 1:10 in fresh THY+P and grown to the mid-log phase at an optical density (OD_600nm_) of 0.6.

Electrocompetent *S. iniae* strains were made by growing the bacteria in THY+P media containing 0.6% sterile glycine overnight. The overnight culture was diluted in fresh THY+P supplemented with 0.6% sterile glycine and grown to OD_600nm_ of 0.3–0.35. The bacteria were harvested by centrifugation (6000 × *g*, 10 min, 4 °C) in a chilled rotor, and resuspended in 40 mL ice-cold sterile electroporation medium (5% glucose, 1 mM MgCl_2_, pH 6.5). The bacterial cells were pelleted and washed twice by centrifugation before being resuspended in 150 μL of ice-cold sterile electroporation medium. The resulting bacterial competent cell suspension in 50 µL was mixed with 1–10 µg purified plasmid and transferred into a prechilled Micropulser™ electroporation cuvette (Bio-Rad, Hercules, CA, USA). A single pulse of 2.1 kV, capacitance at 25 µF and resistance at 200 Ω was applied to the bacterial cell suspension by using a Gene Pulser Xcell™ (Bio-Rad), and 1 mL THY+P was added immediately into the cuvette. The bacterial cell suspension was transferred into an Eppendorf tube, and allowed to recover at 37 °C for 3 h. The bacteria cells were then centrifuged and resuspended in 100 µL THY+P. The resuspended bacterial cells were plated on a THY+P agar plate containing an appropriate amount of antibiotic and incubated at 37 °C overnight.

### 2.2. Allelic Exchange Mutagenesis

The individual *S. iniae spnAi* and *s5nAi* gene-knockout mutants (∆*spnAi* and ∆*s5nAi*) were created by allelic replacement of the target gene on the wild-type (WT) *S. iniae* genome with a spectinomycin resistance gene, *aad9* (Figure S1). Primers used to generate and confirm the allelic exchange are listed in Table 1. Approximately 1000-bp of the upstream flanking region (FR1) and downstream FR2 of the *spnAi* or *s5nAi* genes were amplified by 25 cycles of PCR using iProof™ high-fidelity DNA polymerase (Bio-Rad, Hercules, CA, USA) at an annealing temperature of 56 °C. The amplified fragments were cloned into the multiple cloning sites flanking the *aad9* gene on the pFW11 plasmid [41]. The generated plasmid construct was then transformed into WT *S. iniae* by electroporation, and the successful transformants were selected using a THY+P agar plate containing spectinomycin. The targeted in-frame replacement of *spnAi* and *s5nAi* were confirmed by PCR using the *aad9* forward primer (aad9.fw) and a reverse diagnostic primer (SpnAi_DP.rv for *spnAi* and S5nAi_DP.rv for *s5nAi*) that anneal downstream of the FR2 region.

### 2.3. Green Fluorescence Labelling of Wild-Type S. iniae, Isogenic Mutants and Complementation Strains

Green fluorescent *S. iniae*, *S. iniae* ∆*spnAi* and *S. iniae* ∆*s5nAi* were generated by transformation with the toxin-antitoxin stabilized green fluorescent reporter plasmid pLZ12Km2-P23R:TA:gfpmut2 [42] by electroporation. The generation of green fluorescent complementation strains (*S. iniae* ∆*spnAi*:*spnAi* and *S. iniae* ∆*s5nAi*:*s5nAi*) were achieved by inserting the *spnAi* and *s5nAi* genes into the green fluorescent reporter plasmid and transforming the *S. iniae* ∆*spnAi* and *S. iniae* ∆*s5nAi* deletion strains, respectively. A list of primers used to generate and confirm the complementation strains is provided in Table 1. The full length s*pnAi* gene including its ribosome binding site was amplified from the genome of the WT *S. iniae*, and cloned into pLZ12Km2-P23R:TA:gfpmut2 downstream of the original streptococcal promoter sequence (Figure S2). The resulting reporter plasmid carrying the s*pnAi* or *s5nAi* gene was then introduced into ∆*spnAi* or ∆*s5nAi* by electroporation. These complementation strains simultaneously had their *spnAi* or *s5nAi* genes restored along with green fluorescence reporter. Successful *S. iniae* transformants were first selected on THY+P agar plates containing kanamycin, then screened for the ability to fluoresce.

### 2.4. DNA-Methyl Green Assay

The DNA-methyl green assay [43] was used to quantify the DNase activity of the generated *S. iniae* strains. Bacterial cells (10^6^ colony-forming units, CFU) were resuspended in 50 µL of nuclease reaction buffer (25 mM Tris-HCl, pH 7.0, 1 mM CaCl_2_ and 3 mM MgCl_2_) and transferred into a clear-bottom Corning^®^ 96-well plate (Sigma-Aldrich, St. Louis, MO, USA). An equal volume of DNA-methyl green substrate solution was then added and the plate was incubated at 37 °C for 20 h before measuring the absorbance at A_492nm_ and A_620nm_ using an EnSpire™ 2300 plate reader (Perkin Elmer, Waltham, MA, USA). The corrected absorbance was calculated by subtracting the A_492nm_ reading from the A_620nm_ reading. 

### 2.5. Quantification and Visualization of NETs Destruction

*Tg*(*lyz*:*EGFP*)*^nz117^* adult zebrafish [44] were anaesthetized in 0.168 mg/mL tricaine (Sigma-Aldrich) before dissection of the kidneys. The isolated kidney tissue was pooled and dissociated by gently teasing through a 40 µm Falcon™ cell strainer (BD Biosciences) in 2 mL ice-cold Hank’s balanced salt solution without phenol red (HBSS, Thermo Fisher Scientific, Waltham, MA, USA). The homogenized solution was again passed through the strainer twice and transferred to Falcon™ round-bottom polystyrene tubes (BD Biosciences). The enhanced green fluorescence protein (EGFP) labeled neutrophils were sorted using a FACSAria™ II cell-sorting system (BD Biosciences), and a volume containing 5 × 10^4^ neutrophils in 100 µL HBSS was used in each reaction.

To quantify the NET destruction, the neutrophils were seeded in a solid-bottom Corning^®^ 96-well plate and activated by the addition of phorbol 12-myristate 13-acetate (PMA, Sigma-Aldrich) at a final concentration of 1 µg/mL at 37 °C for 2 h. Cytochalasin D (Sigma-Aldrich) in a final concentration of 2 µg/mL was added to the activated neutrophils before adding 2.5 × 10^6^ bacterial cells. As a positive control, three wells of neutrophils were incubated with 2 U DNase I (Thermo Fisher Scientific). The reaction was incubated at 37 °C for a further 1 h. Sytox Orange (Thermo Fisher Scientific) was added to a final concentration of 0.1 µM and incubated at room temperature for 10 min before reading the fluorescence at excitation/emission at 530/590 nm using an EnSpire™ 2300 plate reader.

To visualize the degradation of NETs, a Nunc^®^ Lab-Tek^®^ Eight-well chamber slide (Sigma-Aldrich) was coated with 300 µL per well of 0.0001% poly-L-lysine (Sigma-Aldrich) at 37 °C for 1 h. Unbound poly-L-lysine was removed by washing once with UltraPure™ water (Invitrogen, Waltham, MA, USA). Each well of the slide was seeded with 5 × 10^4^ neutrophils in 200 µL HBSS, and allowed to settle at 37 °C for 30 min. Neutrophils were then activated by adding PMA at a final concentration of 1 µg/mL and incubated at 37 °C for 2 h. The solution in the well was carefully removed before 2.5 × 10^6^ bacterial cells and Cytochalasin D at a final concentration of 2 µg/mL were added. As a positive control, three wells of neutrophils were incubated with 2 U DNase I. The reaction mixture was incubated at 37 °C for a further 1 h, followed by washing once with HBSS. The slide was fixed with 4% paraformaldehyde (PFA) in phosphate-buffered saline (PBS) at 4 °C for 1 h and washed 3 times with HBSS. The cells were permeabilized with 0.05% Triton X-100 and washed 3 times with HBSS. The slide was blocked with 300 µL of immunostaining blocking solution (1% bovine serum albumin in HBSS) at 4 °C overnight. The blocking solution was removed the next day and the slide was incubated with 200 µL of 1:1000 prediluted rabbit anti-human neutrophil elastase (Sigma-Aldrich) antibody in blocking solution at 37 °C for 1 h, followed by an incubation with 200 µL of 1:500 prediluted goat anti-rabbit IgG FITC (Abacus ALS, Queensland, Australia) in blocking solution at 37 °C for 1 h in the dark. The chamber frame was removed and ProLong^®^ Gold Antifade Mountant containing DAPI (Molecular Probes, Eugene, OR, USA) was added to the slide and a coverslip was gently placed on top of the reagent. The slide was allowed to cure at room temperature for 24 h in the dark before being analyzed using a Nikon Eclipse E600 fluorescence microscope (Nikon, Tokyo, Japan).

### 2.6. Malachite Green Phosphate Colorimetric Assay

The malachite green phosphate colorimetric assay [43] was used to quantify the nucleotidase activity of the modified *S. iniae* strains. Bacterial cells (10^6^ CFU) were resuspended in 98 µL of nucleotidase reaction buffer (50 mM Tris-HCl, pH 7.0 and 10 mM MgCl_2_) and transferred into a clear-bottom Corning^®^ 96-well plate. A final concentration of 1 mM AMP (Sigma-Aldrich) substrate was added and incubated at 37 °C for 30 min. The reactions were stopped by adding EDTA to a final concentration of 50 mM and the release of inorganic phosphate (P_i_) was then quantified using a malachite green phosphate colorimetric assay kit (Sigma-Aldrich) according to the manufacturer’s instruction. The release of P_i_ was measured at A_650nm_ and the amount of P_i_ was calculated based on a standard curve of P_i_.

### 2.7. Zebrafish Maintenance

Adult zebrafish were maintained in the commissioned zebrafish facility at The University of Auckland according to the standard operating procedures (SOP691) of the facility, with approval from the University of Auckland Animal Ethics Committee. The facility has a 14 h light and 10 h dark automated lighting cycle and the water conditions for the zebrafish maintenance are set to between pH 7.2 and 7.5, temperature 25.5 and 29.5 °C and conductivity 250 and 500 µS. Embryos were obtained by natural spawning after light exposure, and were then incubated in E3 medium (5 mM NaCl, 0.17 mM KCl, 0.33 mM CaCl_2_ and 0.33 mM MgCl_2_) with 0.1% (v/v) methylene blue (PanReac AppliChem, Darmstadt, Germany) at 28 °C. At 1 day post-fertilization (dpf), embryos were manually dechorionated. The larvae were then transferred to a new Petri dish containing E3 medium supplemented with 0.003% phenylthiourea (PTU) to prevent pigmentation, and incubated at 28 °C. Wild-type (AB line) zebrafish larvae were used in survival analysis, bacterial load determination and time-lapse confocal imaging experiments. The previously published transgenic lines *Tg*(*lyz*:*DsRED2*)*^nz50^* [44] and *Tg*(*mpeg1*:*EGFP*)*^gl22^* [45] were used in the neutrophil and macrophage recruitment assays, respectively. *Tg*(*lyz*:*DsRED2*)*^nz50^* is a transgenic zebrafish line in which the neutrophil-specific promoter *lyz* drives the neutrophil expression of DsRED2, rendering neutrophils red fluorescent [44]. *Tg*(*mpeg1*:*EGFP*)*^gl22^* is a transgenic zebrafish line in which the macrophage-specific promoter of *macrophage expressed gene 1* (*mpeg1*) drives macrophage expression of EGFP, rendering macrophages green fluorescent [45].

### 2.8. Preparation and Microinjection of S. iniae 

One milliliter of mid-log phase bacterial cultures were centrifuged (2300 × *g*, 10 min, 4 °C) and resuspended in 1 mL sterile PBS. The OD_600nm_ of resuspended bacterial cells was measured again and diluted to OD_600nm_ of 0.1 in 1 mL sterile PBS. The bacterial cells were centrifuged again and resuspended with 0.25% phenol red in sterile PBS to a final concentration of 50 CFU/nL. Two dpf larvae were anesthetized in tricaine before being mounted in 3% methylcellulose (Sigma-Aldrich) in E3 medium. One nanoliter of bacterial suspension was microinjected into the hindbrain (Figure S3) of the larvae. The injection dose was validated by injecting one bolus into 10 µL sterile PBS, spot plated on the THY+P agar plate containing kanamycin and incubated at 37 °C overnight for enumeration. After injection, larvae were then transferred back to the Petri dish containing E3 medium supplemented with PTU and incubated at 28 °C. The larvae survival at indicated time-points was monitored. The determination of live or dead larvae was based on the presence of a heartbeat and response to gentle touching with a sterile transfer pipette.

### 2.9. S. iniae Enumeration From Infected Larvae

At the indicated times, infected larvae were anaesthetized and each larva was collected in a tube. Twenty microliters of sterile dissociation buffer (PBS with 1% Triton-X) was used to homogenize the larva by repeatedly pipetting up and down. For enumeration, serial dilution of the homogenized mixture was made in sterile PBS, spot plated on THY+P agar plate containing kanamycin and incubated at 37 °C overnight.

### 2.10. Mounting and Confocal Imaging of Infected Larvae

Infected larvae were anaesthetized before mounting in 0.8% UltraPure™ low melting point agarose (Invitrogen) in E3 medium supplemented with PTU. Z-Series time-lapse fluorescence images were acquired using a Fluoview FV1000 laser scanning confocal microscope (Olympus, Tokyo, Japan) with a 20× water immersion objective lens. For live cell imaging, the temperature of the incubation chamber was preadjusted to 28 °C. Z-stacks of the hindbrain ventricle in dorsal view (Figure S3, bottom panel) were taken with a total of 36 sections at 5 µm step size intervals every 5 min. Confocal imaging was carried out with the similar acquisition setting in each set of experiments. The resulting images were analyzed using the Volocity 3D image analysis software V6.1.1 (Quorum Technologies, Lewes, UK) and the images were made into movies using ImageJ V1.52k [46].

### 2.11. Immunofluorescence Detection of Innate Immune Cells in Infected Larvae

At the indicated time-points, infected larvae were anaesthetized and collected in a tube. One milliliter of ice-cold fixing solution (4% PFA and 4% sucrose in PBS) was added and incubated at 4 °C overnight. Larvae were dehydrated the next day with increasing successive concentrations of methanol in PBS with 0.1% Tween 20 (PBS-T) for 5 min each incubation and stored at −20 °C for up to a week. Dehydrated larvae were rehydrated with decreasing successive concentrations of methanol in PBS-T for 5 min each incubation and washed once with PBS-T for 5 min before being incubated in 1 mL of ice-cold pure acetone on a shaker for 7 min to allow tissue permeabilization. The larvae were washed with PBS-T for 5 min three times, and incubated in 1 mL of immunostaining blocking solution (10% goat serum, 2% blocking reagent and Roche in maleic acid buffer) on a shaker for 2 h. The immunostaining blocking solution was replaced with 200 µL of 1:500 prediluted primary antibody anti-DsRED2 (Clonetech, Mountain View, CA, USA) for detecting neutrophils, or with anti-GFP (Abcam, Cambridge, UK) for detecting macrophages in immunostaining blocking solution, and incubated on a shaker at 4 °C overnight. The larvae were washed with PBS-T for 5 min four times to remove unbound antibodies before incubated with 100 µL of 1:500 prediluted secondary antibody anti-rabbit IgG-Alexa Fluor 568 (Thermo Fisher Scientific, Waltham, MA, USA) or anti-chicken IgG-Alexa Fluor 488 (Thermo Fisher Scientific, Waltham, MA, USA) in immunostaining blocking solution in the dark on a shaker for 2 h. From this point onwards, the larvae were protected from light exposure to avoid photobleaching of the fluorophores. The larvae were washed with PBS-T again for 5 min four times and fixed with 1 mL ice-cold fixing solution and stored at 4 °C overnight. The larvae were mounted and imaged as described in the previous section.

### 2.12. Statistical Analyses

The statistical analyses were performed using GraphPad Prism V7.03 (GraphPad Software, San Diego, CA, USA). Statistical significance was calculated using either the unpaired two-tailed *t*-test or 2-way ANOVA with a Tukey’s multiple comparisons test. The statistical significance analysis for Kaplan–Meier curves was calculated using the Gehan–Breslow–Wilcoxon test. A *p* value of <0.05 was considered to be statistically significant. *p* value <0.05 was labeled with *, *p* value <0.01 was labeled with **, *p* value <0.001 was labeled with *** and *p* value <0.0001 was labeled with ****. A *p* value of >0.05 was considered not statistically significant and was labeled with ns.

## 3. Results

### 3.1. Deletion of SpnAi Decreases DNase Activity in S. iniae 

**Figure 1:**
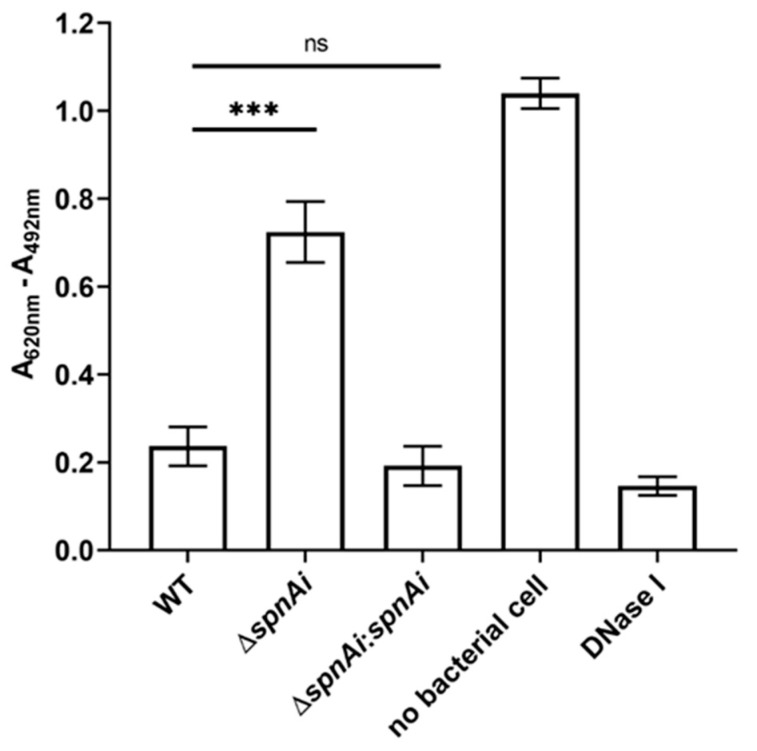
DNase activity of *Streptococcus iniae* strains. The DNase activity against salmon sperm DNA was determined for wild-type (WT) *S. inia*, *S. iniae* ∆*spnAi* and *S. iniae* ∆*spnAi*:*spnAi* using a methyl green assay [43]. A decrease in the reading of A_620nm_ - A_492nm_ indicates DNA digestion. The error bars show the standard deviation of three experiments performed in triplicate. ***, *p* < 0.001; ns, not significant (as determined by an unpaired two-tailed *t*-test).

Our previous study showed that recombinant SpnAi is able to digest DNA in vitro [18]. To confirm that SpnAi also shows activity in vivo, a *spnAi* gene deletion mutant (*S. iniae* ∆*spnAi*) and the corresponding complementation strain (*S. iniae* ∆*spnAi*:*spnAi)* were analyzed for DNase activity using a DNA-methyl green assay. As shown in Figure 1, deletion of the *spnAi* gene resulted in significant loss of DNase activity (approximately 3-fold, *p* < 0.001). Complementation with the *spnAi* gene resulted in a DNase activity level similar to that of the WT *S. iniae* strain. This activity was also similar to 1 U of commercial DNase I.

### 3.2. SpnAi Promotes Degradation of NETs

**Figure 2:**
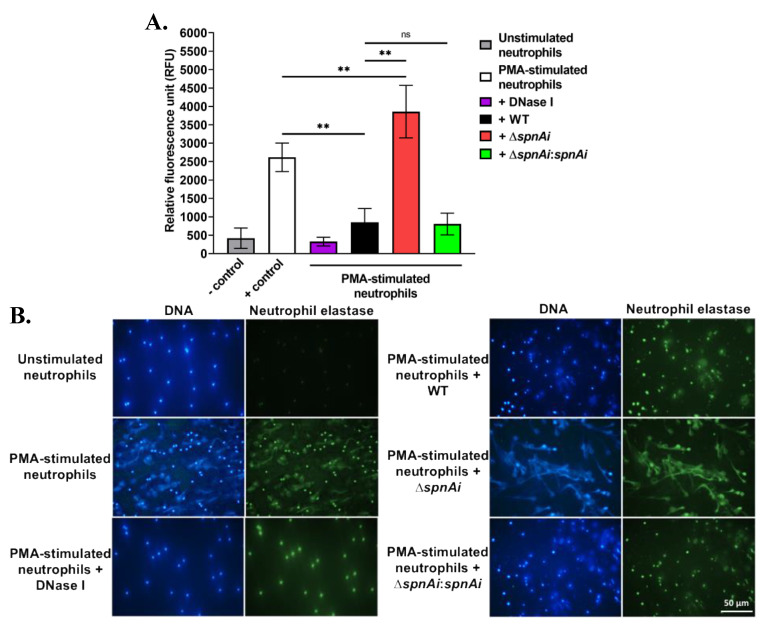
Release and destruction of neutrophil extracellular traps (NETs) by various *S. iniae* strains. (**A**) The extracellular DNA stain Sytox Orange was used to quantify the NETs and is presented as relative fluorescence units (RFU). NETs were degraded in the presence of WT *S. iniae* and DNase I (positive control), but not in the presence of the *spnAi* deletion mutant. Complementation of the mutant strain with a functional *spnAi* gene restored the NET-degrading ability of the mutant strain. Error bars show the standard deviation of two experiments performed in triplicate. **, *p* < 0.01; ns, not significant (as determined by an unpaired two-tailed *t*-test). (**B**) Immunofluorescence microscopy of zebrafish neutrophils. DAPI stain (blue) indicates both the neutrophil nuclei and released NETs. A cross-reactive human antibody against zebrafish neutrophil elastase, a bactericidal enzyme associated with NETs, was used to label the released NETs (green). Activation of unstimulated neutrophils (top left) results in NET release (middle left), which could be degraded by adding DNase I (bottom left). Addition of WT *S. iniae* also led to NETs destruction (top right), which was abrogated in the *spnAi* deletion mutant (middle right) and restored by complementing the deletion mutant with the *spnAi* gene (bottom right).

The destruction of NETs by extracellular nucleases has been demonstrated for other streptococci, including *S. pyogenes* SpnA [16], *S. suis* SsnA [47] and *S. pneumoniae* TatD [48]. The ability of SpnAi to digest the backbone DNA of NETs from fish neutrophils was therefore hypothesized and investigated. Neutrophils were isolated from the kidneys of adult zebrafish and stimulated with PMA for 2.5 h to generate NETs, before being incubated with *S. iniae* WT and mutant strains. DNA released from NETs was quantified using the extracellular DNA stain Sytox Orange (Figure 2A). Incubation of WT *S. iniae* with stimulated neutrophils substantially decreased the fluorescence signal (approximately 3-fold) indicating DNA degradation (*p* < 0.01). DNase I was used as a control and reduced fluorescence approximately 8-fold. Notably, the incubation with the *spnAi* deletion strain not only showed no reduction in NETs degradation, but also increased the amount of extracellular DNA (*p* < 0.01). As expected, incubation with the complementation strain with stimulated neutrophils degraded NETs at a similar level as the WT strain.

The destruction of NETs by various *S. iniae* strains was also visualized using immunofluorescence microscopy (Figure 2B). The extracellular filamentous structures stained for DNA (blue) and neutrophil elastase (green) confirmed the presence of NETs. No NET-like structure was visible in the absence of PMA stimulant, but after PMA stimulation, extracellular structures were apparent. When the activated neutrophils were exposed to *S. iniae* WT or DNase I was added as a positive control, the web-like structures disappeared indicating degradation of NETs. In contrast, when the *S. iniae* ∆*spnAi* mutant was added to the activated neutrophils, the net-like structure remained visible, showing that *spnAi* was involved in the degradation of NETs. Complementation of the mutant strain with a functional *spnAi* gene restored the NET-degrading ability of the mutant strain.

### 3.3. Deletion of S5nAi Decreases Nucleotidase Activity in S. iniae

**Figure 3:**
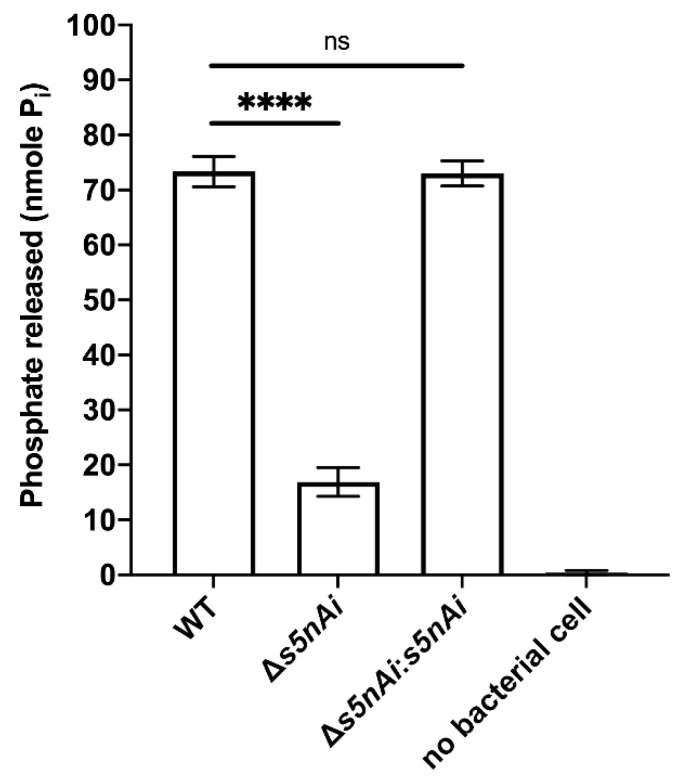
Nucleotidase activity of *S. iniae* strains. WT, ∆*s5nAi* and ∆*s5nAi*:*s5nAi* were incubated with 1 mM AMP as a substrate and the P_i_ released was measured using a malachite green phosphate colorimetric assay. Error bars show the standard deviation of three experiments performed in triplicate. ****, *p* < 0.0001; ns, not significant (as determined by an unpaired two-tailed *t*-test).

We have previously shown that recombinant S5nAi is able to generate adenosine from ADP and AMP [18]. To show that the bacteria produce the active enzyme, a *s5nAi* deletion mutant (∆*s5nAi*) was generated along with a complementation strain (∆*s5nAi*:*s5nAi*). The nucleotidase activity of *S. iniae* WT and the mutant strains was tested by quantifying the amount of P_i_ released from 1 mM AMP (Figure 3). Incubation of WT *S. iniae* resulted in the release of about 70 nmole P_i_ indicating nucleotidase activity. In contrast, AMP hydrolysis with the ∆*s5nAi* deletion mutant resulted in significantly lower amounts of P_i_ (approximately 4.3-fold, *p* < 0.0001). No measurable levels of P_i_ were found when no bacteria were added excluding spontaneous hydrolysis of AMP. Full nucleotidase activity could be restored in the ∆*s5nAi*:*s5nAi* complementation mutant.

### 3.4. SpnAi and S5nAi Contribute to S. iniae Virulence in Zebrafish Larvae

**Figure 4:**
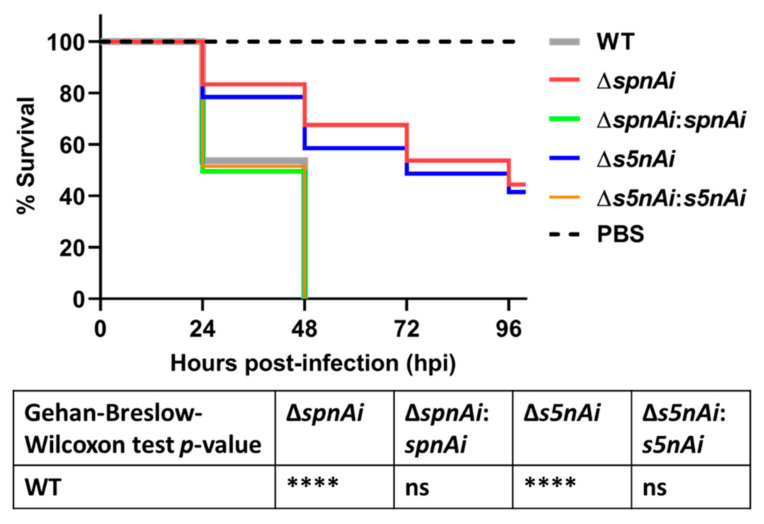
Survival analysis of zebrafish larvae infected with WT and mutant *S. iniae* strains. WT, ∆*spnAi*, ∆*s5nAi* and their complementation strains were each injected into 2 days post-fertilization (dpf) larvae (wild-type AB line). The survival of infected larvae was recorded over a 96 h-period. This data is the combined result from 3 independent experiments (*n* = 35 per group in each experiment). ****, *p* < 0.0001; ns, not significant (as determined by the a Gehan–Breslow–Wilcoxon test).

Next, we investigated the virulence attributes of SpnAi and S5nAi using a zebrafish infection model. The contribution of the two enzymes to *S. iniae* survival in the zebrafish host was analyzed by injecting 2 days post-fertilization (dpf) larvae (wild-type AB line) with 50 CFU of WT and mutant *S. iniae* strains. The survival of larvae was recorded every 24 h over a 96 h time-course (Figure 4). Infection with WT *S. iniae* killed all larvae within 48 h, whereas infection with the deletion mutants, ∆*spnAi* and ∆*s5nAi*, showed increased survival rates 48 h post-infection (hpi) of approximately 63% and 60%, respectively, and about 40% survival at 96 hpi. Zebrafish infected with the complementation strains with *spnAi* and *s5nAi* genes restored, displayed a similar mortality rate as the WT.

### 3.5. SpnAi and S5nAi Support the Growth of S. iniae in Zebrafish Larvae

**Figure 5:**
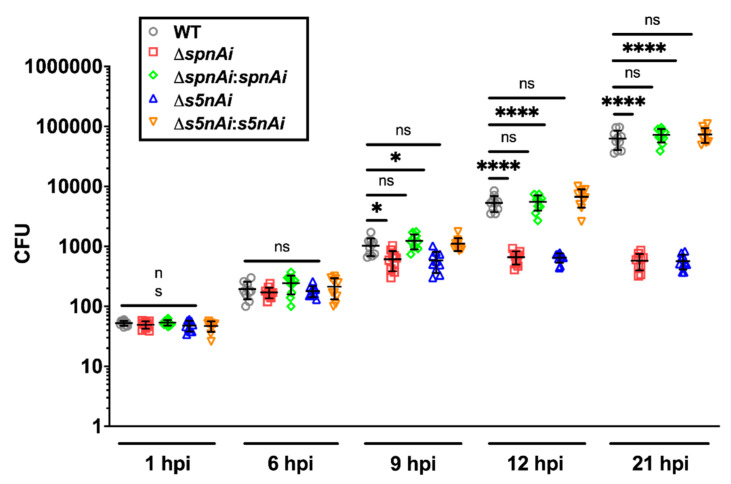
Bacterial load of zebrafish larvae infected with WT and mutant *S. iniae* strains. WT, ∆*spnAi*, ∆*s5nAi* and their complementation strains were each injected into 2 dpf larvae (wild-type AB line). The bacterial load of infected larvae (*n* = 5 per group in each experiment) at indicated time-points were quantified by plating on kanamycin-containing THY+P agar plates. This data is the combined result from 2 independent experiments (*n* = 5 per group in each experiment). The error bars show the standard deviation of two experiments. *, *p* < 0.05; ****, *p* < 0.0001; ns, not significant (as determined by a 2-way ANOVA with a Tukey’s multiple comparisons test).

As a measurement of disease progression, the bacterial load in whole zebrafish larvae (wild-type AB line) were monitored using the number of bacteria recovered from homogenized larvae (Figure 5). The growth rate of the *S. iniae* strains were first tested in vitro in order to investigate whether the deletion of *spnAi* or *s5nAi* on the *S. iniae* WT genome and the complementation of ∆*spnAi* or ∆*s5nAi* would affect their growth compared to their parental strain (Figure S4). Overall, no significant difference was seen between the growth rate of WT, ∆*spnAi* or ∆*s5nAi* and the complementation strains (Figure S4), confirming that the gene deletions and complementations had no effect on bacterial growth.

The disease progression experiment showed that the amount of WT bacteria recovered from infected larvae increased 2000-fold over a 21 h time-course, from an initial inoculum of 50 CFU to 1 × 10^5^ CFU at 21 hpi, which was similar to the bacterial load in larvae infected with *spnAi* and *s5nAi* complementation strains. Single gene-knockout *S. iniae* showed lower bacterial counts, as the number of bacteria recovered from larvae infected with ∆*spnAi* and ∆*s5nAi* mutants increased 20-fold from an initial inoculum of 50 CFU to a maximum of 1000 CFU at 9 hpi that was maintained until the end of the experiment. An additional group of larvae was injected with sterile PBS as a negative control and showed no colony formation on the agar plates (data not shown). These results explained the high mortality rate of larvae infected with WT and complementation strains, and higher survival rate of larvae infected with ∆*spnAi* and ∆*s5nAi* mutants at 24 h.

### 3.6. Wild-type S. iniae, but Not SpnAi and S5nAi Deletion Mutants Were Able to Proliferate and Disseminate in Zebrafish Larvae

**Figure 6:**
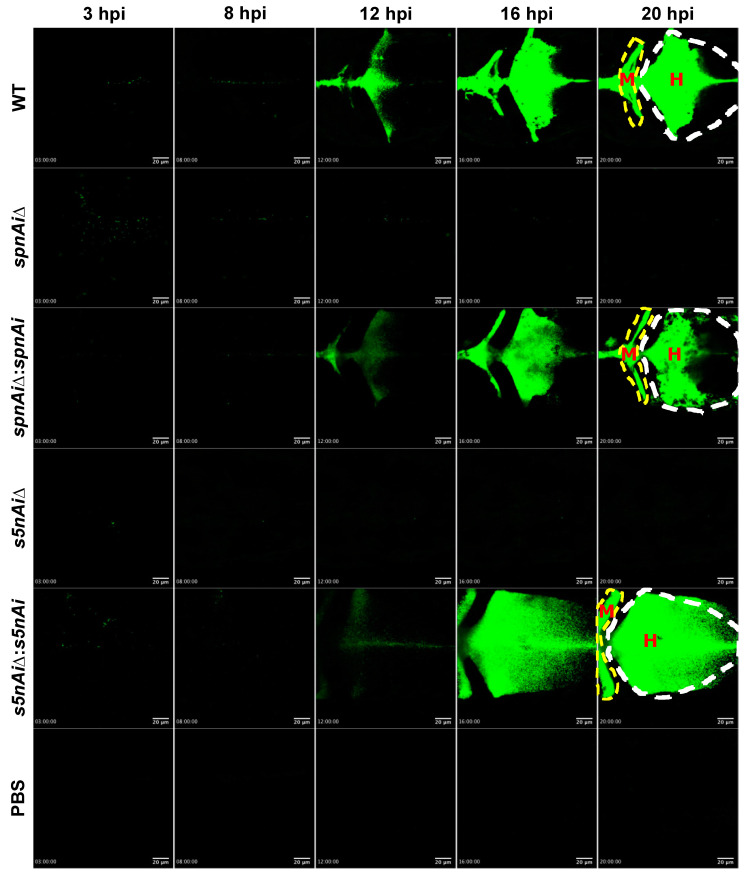
Time-lapse confocal imaging of 2 dpf zebrafish larvae infected with green fluorescent-labeled *S. iniae* or mutant strains. Confocal microscopy was used to observe the proliferation and dissemination of green fluorescent-labeled *S. iniae* at the infection site. Larvae (wild-type AB line) were injected with 50 colony-forming units (CFU) of gfpmut2-labelled WT, ∆*spnAi*, ∆*spnAi*:*spnAi*, ∆*s5nAi*, ∆*s5nAi*:*s5nAi* and sterile PBS at the hindbrain ventricle. Images of the hindbrain ventricle in dorsal view were taken from 3 to 21 hpi using a confocal microscope with a 20× objective lens. Five frames at 3, 8, 12, 16 and 20 hpi were extracted from the individual movies. H, hindbrain ventricle; M, midbrain ventricle. Scale bar, 20 µm. In the supplemental materials, see Movies S1. Results are representative of 2 independent experiments.

To visualize the survival of *S. iniae* in the presence or absence of SpnAi or S5nAi at the site of infection, approximately 50 CFU of green fluorescent-labeled *S. iniae* or mutant strains were individually injected into the hindbrain ventricle of 2 dpf larvae (wild-type AB line). An additional group of larvae were injected with sterile PBS as a control for this experiment. The hindbrain ventricle of each infected or control larvae in dorsal view (Figure S3, bottom panel) was imaged using a confocal microscope from 3 to 21 hpi (Figure 6; see Movies S1 in the supplemental materials). Overall, the area of green fluorescence increased in WT *S. iniae*-infected larvae over the 21 h time-course, particularly starting from 12 hpi (Figure 6). Similar observations were made in larvae infected with ∆*spnAi*:*spnAi* and ∆*s5nAi*:*s5nAi* but not ∆*spnAi* and ∆*s5nAi*. Furthermore, it was also observed that the WT and ∆*spnAi*:*spnAi* and ∆*s5nAi*:*s5nAi* strains disseminated from the hindbrain ventricle (Figure 6, labeled as H) to the midbrain ventricle (Figure 6, labeled as M) of the larvae.

### 3.7. Zebrafish Larvae Infected with S. iniae ∆SpnAi Showed an Increasing Number of Neutrophils at the Site of Infection over Time

**Figure 7:**
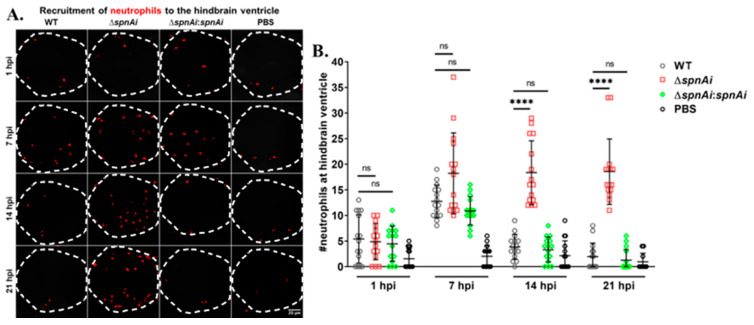
Quantification of neutrophils recruited at the site of *S. iniae* infection. (A) Immunofluorescence detection of neutrophils recruited to the hindbrain ventricle of *Tg*(*lyz:DsRED2*)*^nz5^^0^* larvae infected with 50 CFU of WT (first column), ∆*spnAi* (second column), ∆*spnAi*:*spnAi* (third column) or sterile PBS (last column) at 1 hpi (first row), 7 hpi (second row), 14 hpi (third row) and 21 hpi (last row). The larvae (*n* = 15 per group per time-point) were infected at 2 dpf and fixed at the indicated time-points. A confocal microscope was used to image immunostained neutrophils at the hindbrain ventricle of each larvae in dorsal view (white dotted line). Scale bar, 20 µm. (B) Quantification of neutrophils in the hindbrain ventricle of individual larvae. ****, *p* < 0.0001; ns, not significant (as determined by a 2-way ANOVA with a Tukey’s multiple comparisons test).

SpnAi was shown to digest NETs released from activated neutrophils (Figure 2). Therefore, we were interested to investigate the effect of SpnAi on neutrophil recruitment to the site of infection. In order to quantify the number of neutrophils recruited to the hindbrain ventricle at various time-points after infection, *Tg*(*lyz:DsRED2*)*^nz50^* larvae infected with WT or mutant *S. iniae* strains were fixed at 1, 7, 14 and 21 hpi. An additional group of larvae were injected with sterile PBS as a control for this experiment. Neutrophils were rendered red by immunostaining, and neutrophils recruited to the hindbrain ventricle were quantified by confocal microscopy (Figure 7). The results showed a slight increase in the number of neutrophils recruited to the *S. iniae* infection site as compared to control larvae at 1 hpi, indicating that neutrophils responded to *S. iniae* infection immediately at the hindbrain ventricle. At 7 hpi, the number of neutrophils recruited to the hindbrain ventricle of infected larvae continued to increase with no significant difference in the neutrophils number between the groups of *S. iniae* infected larvae. A significant difference in the number of neutrophils at the hindbrain ventricles was seen between larvae infected with WT and ∆*spnAi* at 14 hpi and 21 hpi (*p* < 0.0001). The number of neutrophils at the hindbrain ventricle of larvae infected with WT and ∆*spnAi*:*spnAi* was significantly lower as compared to ∆*spnAi* at 14 hpi and 21 hpi. 

### 3.8. Zebrafish Larvae Infected with S. iniae ∆S5nAi Showed an Increasing Number of Macrophages at the Site of Infection over Time

**Figure 8:**
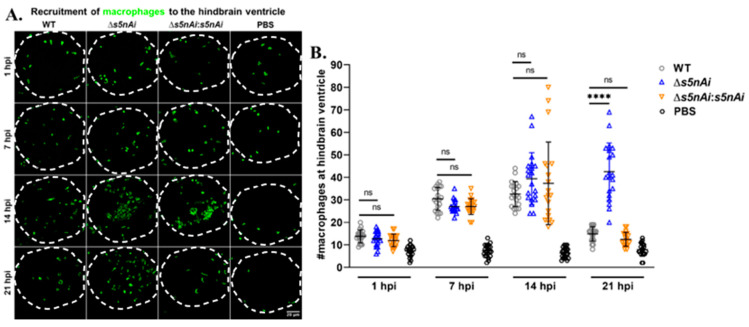
Quantification of macrophages recruited to the site of *S. iniae* infection. (A) Immunofluorescence detection of macrophage recruited to the hindbrain ventricle of *Tg*(*mpeg1:EGFP*)*^gl22^* larvae infected with 50 CFU of WT (first column), Δ*s5nAi* (second column), ∆*s5nAi*:*s5nAi* (third column) or sterile PBS (last column) at 1 hpi (first row), 7 hpi (second row), 14 hpi (third row) and 21 hpi (last row). The larvae (*n* = 20 per group per time-point) were infected at 2 dpf and fixed at indicated time-points. Confocal microscopy was used to image immunostained macrophages at the hindbrain ventricle of each larvae in the dorsal view (white dotted line). Scale bar, 20 μm. (B) Quantification of macrophages in the hindbrain ventricle of individual larvae. An increase in the number of macrophages recruited to the hindbrain ventricle of infected larvae was seen compared to larvae injected with sterile PBS. The number of macrophages at the infection site for all infected larvae peaked at 14 hpi. At 21 hpi, the number of macrophages recruited to the infection site of larvae infected with WT and ∆*s5nAi*:*s5nAi* was significantly lower as compared to Δ*s5nAi*. ****, *p* < 0.0001; ns, not significant (as determined by a 2-way ANOVA with a Tukey’s multiple comparisons test).

We have previously shown that S5nAi is able to convert dAMP into dAdo [18], which is known to be cytotoxic for macrophages [49]. Therefore, we analyzed the in vivo function of S5nAi in relation to the recruitment of macrophages to the infection site. The number of macrophages recruited to the hindbrain ventricle of *Tg*(*mpeg1:EGFP*)*^gl22^* larvae infected with WT or mutant strains at various time-points post-infection was quantified by immunostaining and confocal microscopy (Figure 8). During the first hour post-infection, an increase in the number of macrophages recruited to the infection site was observed in comparison to control larvae, but no significant difference was seen between all infected larvae. As the time progressed, the number of macrophages at the infection site increased as seen at 7 hpi and 14 hpi for all infected larvae. No significant difference in the number of macrophages recruited to the hindbrain ventricle of all infected larvae was observed up to 14 hpi, though at 21 hpi the number of macrophages decreased in larvae infected with WT and ∆*s5nAi*:*s5nAi*, but not with ∆*s5nAi*.

## 4. Discussion

*S. iniae* is a pathogenic and zoonotic bacterium that threatens both human health and aquaculture industries. Numerous *S. iniae* virulence factors have been identified with the advances in molecular biology techniques. Elucidating the function of these virulence factors in *S. iniae* pathogenesis is essential to control infection and for identifying effective vaccine targets. We have recently reported the functional characterization of recombinant forms of two novel *S. iniae* virulence factors; an extracellular nuclease (SpnAi) and a secreted 5′-nucleotidase (S5Ai) [18]. Here, we have shown that both enzymatic activities can be found in live *S. iniae*. Furthermore, both activities could be reduced by deletion of the corresponding genes in the *S. iniae* genome by allelic replacement, confirming the function previously evaluated using recombinant proteins [18].

Deletion of *spnAi* in the *S. iniae* strain 9117 did not completely inactivate DNase activity suggesting that *S. iniae* is able to produce other DNase(s). This has also been reported in other Gram-positive cocci such as the production of Nuc1 [50] and Nuc2 [51] by *S. aureus*, and SpnA [16,52] and Sda1 [53] by *S. pyogenes*. However, the major decrease of *S. iniae* DNase activity seen with the *spnAi* deletion mutant suggests that SpnAi is the main enzyme responsible for the nuclease activity. Like other streptococcal DNases, such as EndA of *S. pneumoniae* [21], SpnA of *S. pyogenes* [16] and SsnA of *S. suis* [47], SpnAi also cleaves NETs released from neutrophils, confirming a role as an immune evasion virulence factor. Notably, the deletion of *spnAi* not only showed the absence of NET degradation activity, but also an increase in the production of NETs when the deletion strain was added to neutrophils. This is expected as known triggers of NET release (NETosis), include PMA, lipopolysaccharide and living/dead bacterial cells [54]. Individual stimulus triggers diverse NETosis pathways, and it has recently been shown that *Streptococcus agalactiae* uses a similar NETosis pathway to PMA [55].

The role in virulence and immune evasion of these novel virulence factors was further highlighted in a zebrafish infection model, which has been widely used to investigate *S. iniae* [56] and other pathogenic bacteria [57,58,59]. Advantages of this infection model include a fully sequenced genome showing significant homology with the human genome [60,61], rapid breeding and transparency of larvae during early life stages. The transparency of the larvae allows time-lapse imaging of biological and disease processes to be carried out. Zebrafish infection studies have shown that both neutrophils and macrophages play important roles in the innate immune response against *S. iniae* [56,62]. However, most infection studies with *S. iniae* uses wild-type bacteria, which are not visible using standard imaging methods. A previous study establishing a *S. iniae*-zebrafish infection model used the CellTracker Red CMPTX dye (Thermo Fisher Scientific, Waltham, MA, USA) for staining, but reported the inability to reliably track *S. iniae* in vivo because the dye became diluted or disappeared as bacteria divided [56]. Furthermore, the same study was unable to accurately measure the *S. iniae* burden in the host since non-selective agar plates were used [56], which allowed the growth of other Gram-positive bacteria that were present as part of the normal zebrafish gut microbiota [63]. Here, we reported for the first time the stable green fluorescence labeling of *S. iniae*, by introducing a novel toxin-antitoxin (TA) stabilized green fluorescent reporter plasmid [42,64,65]. The fluorescent labeling of *S. iniae* enables the reliable and non-invasive tracking of *S. iniae* in vivo, due to the post-segregational killing of those bacteria that have lost the plasmid. In addition, the reporter plasmid also contained a kanamycin resistance gene that allowed for the selective growth of *S. iniae*.

In vivo survival analysis using the zebrafish larvae infection model demonstrated a significant reduction in the virulence of the *S. iniae* Δ*spnAi* and ∆*s5nAi* mutants compared to the parental strain. The reintroduction of the wild-type genes into the *S. iniae* gene deletion strains effectively restored the enzymatic activity, suggesting that gene deletion did not cause any down-stream effects. The attenuated virulence of *S. iniae* Δ*spnAi* and ∆*s5nAi* in the larval zebrafish model was also evident in the relatively constant bacterial load, compared to the WT *S. iniae* and the complementation strains where an increase in abundance was seen in the first 21 hpi. These results corroborate the confocal time-lapse imaging, where increased bacterial load was seen throughout the 21 hpi in larvae infected with *S. iniae* WT and complementation strains, but not the ∆*spnAi* and ∆*s5nAi* mutants. These results suggest that WT *S. iniae* was able to escape from killing by innate immune cells, allowing the bacterium to multiply at the infection site and disseminate to the midbrain of the larvae or even to other parts of the body. These results again indicate that SpnAi and S5nAi play an important role in contributing to *S. iniae* virulence.

Notably, the number of recoverable *S. iniae* ∆*spnAi* and ∆*s5nAi* bacteria from the whole larvae was maintained at 1000 CFU at 9–21 hpi, which was higher in contrast to the number of ∆*spnAi* and ∆*s5nAi* mutants that were rapidly cleared from the hindbrain ventricle. This indicates that some *S. iniae* ∆*spnAi* and ∆*s5nAi* mutants were able to somehow survive and disseminate from the infection site to other parts of the larvae, but not proliferate. It has previously been shown that *S. iniae* is able to survive intracellularly in macrophages of mice [66] and salmon [67], thereby providing an opportunity for bacterial survival and dissemination throughout the host. However, any details of this proposed mechanism in vivo have yet to be elucidated.

To take further advantage of the optically transparent properties of larval zebrafish, the transgenic zebrafish strain *Tg*(*lyz:DsRED2*)*^nz50^* possessing red fluorescent neutrophils were used to investigate the in vivo function of SpnAi. There was no detectable difference between WT and mutant strains in stimulating infiltration of neutrophils to the site of infection at 1 hpi and 7 hpi. Based on our in vitro results, it is possible that destruction of NETs plays an important role in the low neutrophil phagocytosis rate of WT *S. iniae*. Notably, the number of neutrophils at the site of infection decreased at 14 hpi with WT, but not the *spnAi* deletion mutant suggesting an additional function of SpnAi apart from nuclease activity. We have recently shown that *S. pyogenes* SpnA catalytic site mutants lacking nuclease activity were still virulent in a *Galleria mellonella* (wax worm) infection model indicating a nuclease-independent virulence mechanism [68]. Like SpnA [16], SpnAi consists of two large domains, a *C*-terminal nuclease domain and a *N*-terminal domain of unknown function. Further evaluations are required to decipher a potential role of the *N*-terminal domain in prevention of neutrophil recruitment or induction of neutrophil death.

It has previously been reported that adenosine synthase A (AdsA), a secreted 5′-nucleotidase expressed by *S. aureus*, generates dAdo from dAMP, which causes apoptotic killing of macrophages [31]. We have therefore used a transgenic zebrafish strain *Tg*(*mpeg1:EGFP*)*^gl22^* that possesses green fluorescent macrophages to investigate the in vivo function of S5nAi. Similar to the effect of SpnAi on neutrophils, no difference in macrophage recruitment was detected at 7 hpi between WT and *s5nAi* mutant strains. However, WT *S. iniae*, but not the ∆*s5nAi* mutant, reduced macrophage infiltration at 21 hpi. This is in line with previous observations that AdsA prevented macrophage infiltration into staphylococcal abscesses in a mouse infection model due to apoptotic killing mediated by dAdo [69]. S5nAi might have a similar role and the delayed effect may be due to a slow decay of the fluorescent signal after macrophage killing.

A number of virulence factors were discovered following the complete sequencing of the *S. iniae* 9117 genome [8]. In addition to SpnAi and S5nAi, many other documented *S. iniae* virulence factors are also homologous to those expressed by the important solely human pathogen, *S. pyogenes* [5], such as α-enolase [70], streptolysin S [14] and the hyaluronic acid capsule [11] that showed 97%, 73% and 71% in amino acid sequence identity to *S. iniae*, respectively. Therefore, the establishment of a *S. iniae*-zebrafish infection model with the use of fluorescently labeled *S. iniae* in this study also allows for modeling homologues of *S. pyogenes* virulence factors in a natural host system. This would therefore also aid the discovery and exploration of functional relationships of streptococcal virulence factors.

## 5. Conclusions

We have shown that the previously reported in vitro enzymatic activities of recombinant *S. iniae* SpnAi and S5nAi are responsible for virulence traits in *S. iniae* using zebrafish infection models. Our work further establishes SpnAi and S5nAi as important immune evasion factors that might be potential targets for the development of therapeutic agents or vaccines against *S. iniae* infections, both in the aquaculture industry and in human health.

## Figures and Tables

**Table 1:** Primers used in this study. The restriction site in each primer is underlined.

Primer Name	Sequence (5′–3′)
(a) Primers used to generate and confirm ∆*spnAi* and ∆*s5nAi*	
SpnAi_FR1.fw	GCCGGATCCGCAGGCCAATTATCTCTTAG
SpnAi_FR1.rv	CTAAGCTTATTGTAACAGAAACATCAGTC
SpnAi_FR2.fw	CATGCCATGGCACGCCGGAGAAACTTCTG
SpnAi_FR2.rv	CCCCCCGGGAACGGACCACGATGCCAC
S5nAi_FR1.fw	GCCGCTAGCGAAAACCATCAAGGCTTCAACG
S5nAi_FR1.rv	GGCGAGCTCCCGTGGAAATCGTTGACACC
S5nAi_FR2.fw	CATGCCATGGGCCAAAACAAGCACAATGG
S5nAi_FR2.rv	GCTGAATTCGCTTAATGAAGAGCATGCG
aad9.fw	CCTTATTGGTACTTACATGTTTG
SpnAi_DP.rv	GACACTGAACAGGCCTTGGCTG
S5nAi_DP.rv	GTCGATTAAGGCTGATTTAGCC
(b) Primers used to generate ∆*spnAi*:*spnAi* and ∆*s5nAi*:*s5nAi*	
SpnAi_FL_ORF.fw	CAGGATCCTAAAGGAGTTTTTATGTTAAAC
SpnAi_FL_ORF.rv	CGGAATTCTTAGTTTTTTTGACCTTTACG
S5nAi_FL_ORF.fw	CGGGATCCATTAGGAGTTTATATGAAAAAGC
S5nAi_FL_ORF.rv	CGGAATTCTTAGTTTTCTTCTTTTTTCTTGC

## Supplementary Materials

The following are available online at https://www.mdpi.com/2076-2607/8/9/1361/s1, Figure S1: Allelic exchange mutagenesis of *spnAi* and *s5nAi*, Figure S2: Plasmid construction for the generation of green fluorescent *S. iniae spnAi* and *s5nAi* complementation strains, Figure S3: Lateral and dorsal view of a 2 dpf zebrafish larva, Figure S4: Growth rates of *S. iniae* strains. Movie S1: Time-lapse confocal imaging of 2 dpf zebrafish larvae infected with stably green fluorescent labeled *S. iniae* or mutant strains.

## References

[1] Pier G.B., Madin S.H. (1976). Streptococcus iniae sp. nov., a beta-hemolytic streptococcus isolated from an Amazon. freshwater dolphin, Inia geoffrensis. Int. J. Syst. Evol. Microbiol..

[2] Agnew W., Barnes A.C. (2007). Streptococcus iniae: An aquatic pathogen of global veterinary significance and a challenging candidate for reliable vaccination. Vet. Microbiol..

[3] Shoemaker C.A., Klesius P.H., Evans J.J. (2001). Prevalence of Streptococcus iniae in tilapia, hybrid striped bass, and channel catfish on commercial fish farms in the United States. Am. J. Vet. Res..

[4] Lau S.K., Woo P.C., Luk W.-k., Fung A.M., Hui W.-t., Fong A.H., Chow C.-w., Wong S.S., Yuen K.-y. (2006). Clinical isolates of Streptococcus iniae from Asia are more mucoid and β-hemolytic than those from North. America. Diagn. Microbiol. Infect. Dis..

[5] Baiano J.C., Barnes A.C. (2009). Towards control of Streptococcus iniae. Emerg. Infect. Dis..

[6] McMillan D.J., Sanderson-Smith M.L., Smeesters P.R., Sriprakash K.S. (2012). Molecular Markers for the Study of Streptococcal Epidemiology. Host-Pathogen Interactions in Streptococcal Diseases.

[7] Sun J.-R., Yan J.-C., Yeh C.-Y., Lee S.-Y., Lu J.-J. (2007). Invasive infection with Streptococcus iniae in Taiwan. J. Med. Microbiol..

[8] Richards V.P., Palmer S.R., Bitar P.D.P., Qin X., Weinstock G.M., Highlander S.K., Town C.D., Burne R.A., Stanhope M.J. (2014). Phylogenomics and the dynamic genome evolution of the genus Streptococcus. Genome Biol. Evol..

[9] Baiano J.C., Tumbol R.A., Umapathy A., Barnes A.C. (2008). Identification and molecular characterisation of a fibrinogen binding protein from Streptococcus iniae. BMC Microbiol..

[10] Locke J.B., Aziz R.K., Vicknair M.R., Nizet V., Buchanan J.T. (2008). Streptococcus iniae M-like protein contributes to virulence in fish and is a target for live attenuated vaccine development. PLoS ONE.

[11] Locke J.B., Colvin K.M., Datta A., Patel S.K., Naidu N.N., Neely M.N., Nizet V., Buchanan J.T. (2006). Streptococcus iniae Capsule Impairs Phagocytic Clearance and Contributes to Virulence in Fish. J. Bacteriol..

[12] Lowe B.A., Miller J.D., Neely M.N. (2007). Analysis of the polysaccharide capsule of the systemic pathogen Streptococcus iniae and its implications in virulence. Infect. Immun..

[13] Miller J.D., Neely M.N. (2005). Large-scale screen highlights the importance of capsule for virulence in the zoonotic pathogen Streptococcus iniae. Infect. Immun..

[14] Bolotin S., Fuller J.D., Bast D.J., de Azavedo J.C. (2007). The two-component system sivS/R regulates virulence in Streptococcus iniae. FEMS Immunol. Med. Microbiol..

[15] Fuller J.D., Camus A.C., Duncan C.L., Nizet V., Bast D.J., Thune R.L., Low D.E., de Azavedo J.C. (2002). Identification of a streptolysin S-associated gene cluster and its role in the pathogenesis of Streptococcus iniae disease. Infect. Immun..

[16] Chang A., Khemlani A., Kang H., Proft T. (2011). Functional analysis of Streptococcus pyogenes nuclease A (SpnA), a novel group A streptococcal virulence factor. Molec. Microbiol..

[17] Zheng L., Khemlani A., Lorenz N., Loh J.M., Langley R.J., Proft T. (2015). Streptococcal 5′-Nucleotidase A (S5nA), a novel Streptococcus pyogenes virulence factor that facilitates immune evasion. J. Biol. Chem..

[18] Soh K.Y., Loh J.M.S., Proft T. (2018). Orthologues of Streptococcus pyogenes nuclease A (SpnA) and Streptococcal 5′-nucleotidase A (S5nA) found in Streptococcus iniae. J. Biochem..

[19] Berends E.T., Horswill A.R., Haste N.M., Monestier M., Nizet V., von Köckritz-Blickwede M. (2010). Nuclease expression by Staphylococcus aureus facilitates escape from neutrophil extracellular traps. J. Innate Immun..

[20] Buchanan J.T., Simpson A.J., Aziz R.K., Liu G.Y., Kristian S.A., Kotb M., Feramisco J., Nizet V. (2006). DNase expression allows the pathogen group A Streptococcus to escape killing in neutrophil extracellular traps. Curr. Biol..

[21] Beiter K., Wartha F., Albiger B., Normark S., Zychlinsky A., Henriques-Normark B. (2006). An endonuclease allows Streptococcus pneumoniae to escape from neutrophil extracellular traps. Curr. Biol..

[22] Brinkmann V., Reichard U., Goosmann C., Fauler B., Uhlemann Y., Weiss D.S., Weinrauch Y., Zychlinsky A. (2004). Neutrophil extracellular traps kill bacteria. Science.

[23] Cekic C., Linden J. (2016). Purinergic regulation of the immune system. Nat. Rev. Immun..

[24] Kumar V., Sharma A. (2009). Adenosine: An endogenous modulator of innate immune system with therapeutic potential. Eur. J. Pharm..

[25] Hasko G., Szabo C., Nemeth Z.H., Kvetan V., Pastores S.M., Vizi E.S. (1996). Adenosine receptor agonists differentially regulate IL-10, TNF-alpha, and nitric oxide production in RAW 264.7 macrophages and in endotoxemic mice. J. Immun..

[26] Cronstein B.N., Kramer S.B., Weissmann G., Hirschhorn R. (1983). Adenosine: A physiological modulator of superoxide anion generation by human neutrophils. J. Exp. Med..

[27] Xaus J., Mirabet M., Lloberas J., Soler C., Lluis C., Franco R., Celada A. (1999). IFN-γ up-regulates the A_2B_ adenosine receptor expression in macrophages: A mechanism of macrophage deactivation. J. Immun..

[28] Bouma M.G., Jeunhomme T., Boyle D.L., Dentener M.A., Voitenok N.N., Van den Wildenberg F., Buurman W.A. (1997). Adenosine inhibits neutrophil degranulation in activated human whole blood: Involvement of adenosine A2 and A3 receptors. J. Immun..

[29] Soh K.Y., Loh J.M., Proft T. (2019). Cell wall-anchored 5′-nucleotidases in Gram-positive cocci. Mol. Microbiol..

[30] Thammavongsa V., Schneewind O., Missiakas D.M. (2011). Enzymatic properties of Staphylococcus aureus adenosine synthase (AdsA). BMC Biochem..

[31] Thammavongsa V., Missiakas D.M., Schneewind O. (2013). Staphylococcus aureus degrades neutrophil extracellular traps to promote immune cell death. Science.

[32] Fan J., Zhang Y., Chuang-Smith O.N., Frank K.L., Guenther B.D., Kern M., Schlievert P.M., Herzberg M.C. (2012). Ecto-5′-nucleotidase: A candidate virulence factor in Streptococcus sanguinis experimental endocarditis. PLoS ONE.

[33] Liu P., Pian Y., Li X., Liu R., Xie W., Zhang C., Zheng Y., Jiang Y., Yuan Y. (2014). Streptococcus suis adenosine synthase functions as an effector in evasion of PMN-mediated innate immunity. J. Infect. Dis..

[34] Dai J., Lai L., Tang H., Wang W., Wang S., Lu C., Yao H., Fan H., Wu Z. (2018). Streptococcus suis synthesizes deoxyadenosine and adenosine by 5′-nucleotidase to dampen host immune responses. Virulence.

[35] Herbomel P., Thisse B., Thisse C. (1999). Ontogeny and behaviour of early macrophages in the zebrafish embryo. Blood.

[36] Hermann A.C., Millard P.J., Blake S.L., Kim C.H. (2004). Development of a respiratory burst assay using zebrafish kidneys and embryos. J. Immunol. Methods.

[37] Le Guyader D., Redd M.J., Colucci-Guyon E., Murayama E., Kissa K., Briolat V., Mordelet E., Zapata A., Shinomiya H., Herbomel P. (2008). Origins and unconventional behavior of neutrophils in developing zebrafish. Blood.

[38] Davidson A.J., Zon L.I. (2004). The ‘definitive’(and ‘primitive’) guide to zebrafish hematopoiesis. Oncogene.

[39] Lam S., Chua H., Gong Z., Lam T., Sin Y. (2004). Development and maturation of the immune system in zebrafish, Danio rerio: A gene expression profiling, in situ hybridization and immunological study. Dev. Comp. Immunol..

[40] Trede N.S., Langenau D.M., Traver D., Look A.T., Zon L.I. (2004). The use of zebrafish to understand immunity. Immunity.

[41] Podbielski A., Spellerberg B., Woischnik M., Pohl B., Lütticken R. (1996). Novel series of plasmid vectors for gene inactivation and expression analysis in group A streptococci (GAS). Gene.

[42] Loh J.M., Proft T. (2013). Toxin–antitoxin-stabilized reporter plasmids for biophotonic imaging of Group A streptococcus. Appl. Microbiol. Biotechnol..

[43] Sinicropi D., Baker D.L., Prince W.S., Shiffer K., Shak S. (1994). Colorimetric determination of DNase I activity with a DNA-methyl green substrate. Anal. Biochem..

[44] Hall C., Flores M.V., Storm T., Crosier K., Crosier P. (2007). The zebrafish lysozyme C promoter drives myeloid-specific expression in transgenic fish. BMC Dev. Biol..

[45] Ellett F., Pase L., Hayman J.W., Andrianopoulos A., Lieschke G.J. (2011). mpeg1 promoter transgenes direct macrophage-lineage expression in zebrafish. Blood.

[46] Schneider C.A., Rasband W.S., Eliceiri K.W. (2012). NIH Image to ImageJ: 25 years of image analysis. Nat. Methods.

[47] de Buhr N., Neumann A., Jerjomiceva N., von Köckritz-Blickwede M., Baums C.G. (2014). Streptococcus suis DNase SsnA contributes to degradation of neutrophil extracellular traps (NETs) and evasion of NET-mediated antimicrobial activity. Microbiology.

[48] Jhelum H., Sori H., Sehgal D. (2018). A novel extracellular vesicle-associated endodeoxyribonuclease helps Streptococcus pneumoniae evade neutrophil extracellular traps and is required for full virulence. Sci. Rep..

[49] Fox I.H., Kelley W.N. (1978). The role of adenosine and 2’-deoxyadenosine in mammalian cells. Annu. Rev. Biochem..

[50] Tucker P.W., Hazen E.E., Cotton F.A. (1978). Staphylococcal nuclease reviewed: A prototypic study in contemporary enzymology. I isolation; physical and enzymatic properties. Mol. Cell. Biochem..

[51] Hu Y., Meng J., Shi C., Hervin K., Fratamico P.M., Shi X. (2013). Characterization and comparative analysis of a second thermonuclease from Staphylococcus aureus. Microbiol. Res..

[52] Hasegawa T., Minami M., Okamoto A., Tatsuno I., Isaka M., Ohta M. (2010). Characterization of a virulence-associated and cell-wall-located DNase of Streptococcus pyogenes. Microbiology.

[53] Aziz R.K., Ismail S.A., Park H.W., Kotb M. (2004). Post-proteomic identification of a novel phage-encoded streptodornase, Sda1, in invasive M1T1 Streptococcus pyogenes. Mol. Microbiol..

[54] Hoppenbrouwers T., Autar A.S., Sultan A.R., Abraham T.E., van Cappellen W.A., Houtsmuller A.B., van Wamel W.J., van Beusekom H.M., van Neck J.W., de Maat M.P. (2017). In vitro induction of NETosis: Comprehensive live imaging comparison and systematic review. PLoS ONE.

[55] Kenny E.F., Herzig A., Krüger R., Muth S., Mondal A., Thompson P.R., Brinkmann V., Von Bernuth H., Zychlinsky A. (2017). Diverse stimuli engage different neutrophil extracellular trap pathways. Elife.

[56] Harvie E.A., Green J.M., Neely M.N., Huttenlocher A. (2013). Innate immune response to Streptococcus iniae infection in zebrafish larvae. Infect. Immun..

[57] Neely M.N., Pfeifer J.D., Caparon M. (2002). Streptococcus-zebrafish model of bacterial pathogenesis. Infect. Immun..

[58] Astin J., Keerthisinghe P., Du L., Sanderson L., Crosier K., Crosier P., Hall C. (2017). Innate immune cells and bacterial infection in zebrafish. Methods Cell Biol..

[59] Miller J.D., Neely M.N. (2004). Zebrafish as a model host for streptococcal pathogenesis. Acta Trop..

[60] Shin J.T., Priest J.R., Ovcharenko I., Ronco A., Moore R.K., Burns C.G., MacRae C.A. (2005). Human-zebrafish non-coding conserved elements act in vivo to regulate transcription. Nucleic Acids Res..

[61] Howe K., Clark M.D., Torroja C.F., Torrance J., Berthelot C., Muffato M., Collins J.E., Humphray S., McLaren K., Matthews L. (2013). The zebrafish reference genome sequence and its relationship to the human genome. Nature.

[62] Vincent W.J., Harvie E.A., Sauer J.-D., Huttenlocher A. (2017). Neutrophil derived LTB4 induces macrophage aggregation in response to encapsulated Streptococcus iniae infection. PLoS ONE.

[63] Roeselers G., Mittge E.K., Stephens W.Z., Parichy D.M., Cavanaugh C.M., Guillemin K., Rawls J.F. (2011). Evidence for a core gut microbiota in the zebrafish. ISME J..

[64] Loh J.M., Proft T. (2016). Stabilized plasmid-based system for bioluminescent labeling of multiple streptococcal species. Biotechnol. Lett..

[65] Cormack B.P., Valdivia R.H., Falkow S.J.G. (1996). FACS-optimized mutants of the green fluorescent protein (GFP). Gene.

[66] Allen J.P., Neely M.N. (2011). The Streptococcus iniae transcriptional regulator CpsY is required for protection from neutrophil-mediated killing and proper growth in vitro. Infect. Immun..

[67] Zlotkin A., Chilmonczyk S., Eyngor M., Hurvitz A., Ghittino C., Eldar A. (2003). Trojan horse effect: Phagocyte-mediated Streptococcus iniae infection of fish. Infect. Immun..

[68] Chalmers C., Khemlani A., Sohn C.R., Loh J.M., Proft T. (2020). Streptococcus pyogenes nuclease A (SpnA) mediated virulence does not exclusively depend on nuclease activity. J. Microbiol. Immunol. Infect..

[69] Thammavongsa V., Kern J.W., Missiakas D.M., Schneewind O. (2009). Staphylococcus aureus synthesizes adenosine to escape host immune responses. J. Exp. Med..

[70] Kim M.S., Choi S.H., Lee E.H., Nam Y.K., Kim S.K., Kim K.H. (2007). α-enolase, a plasmin (ogen) binding and cell wall associating protein from a fish pathogenic Streptococcus iniae strain. Aquaculture.

