# Supplementary material for: Functional Analysis of Two Novel Streptococcus iniae Virulence Factors Using a Zebrafish Infection Model
Source: Microorganisms. 2020 Sep 5;8(9):1361. doi: 10.3390/microorganisms8091361 (PMC7564053; doi:10.3390/microorganisms8091361)
Supplement: Supplementary file 1 [file microorganisms-08-01361-s001.zip › microorganisms-919652-supplementary-update.pdf]

Figure S1.

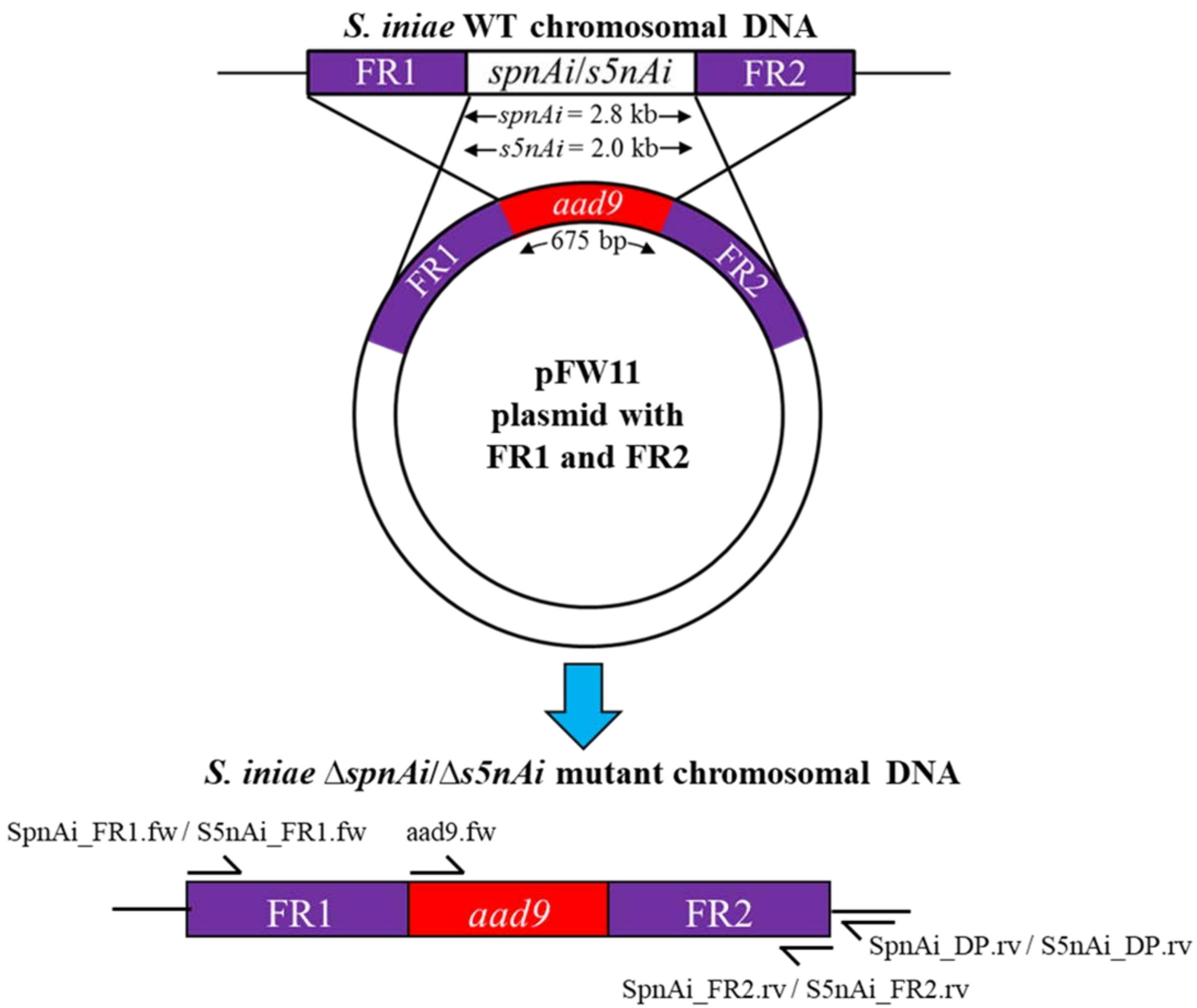

Figure S1. Allelic exchange mutagenesis of *spnAi* and *s5nAi*. The double crossover homologous recombination occurred when the constructed plasmid was introduced into WT *S. iniae* by electroporation, resulting in the replacement of *spnAi* or *s5nAi* with *aad9* in the genome.

Figure S2.

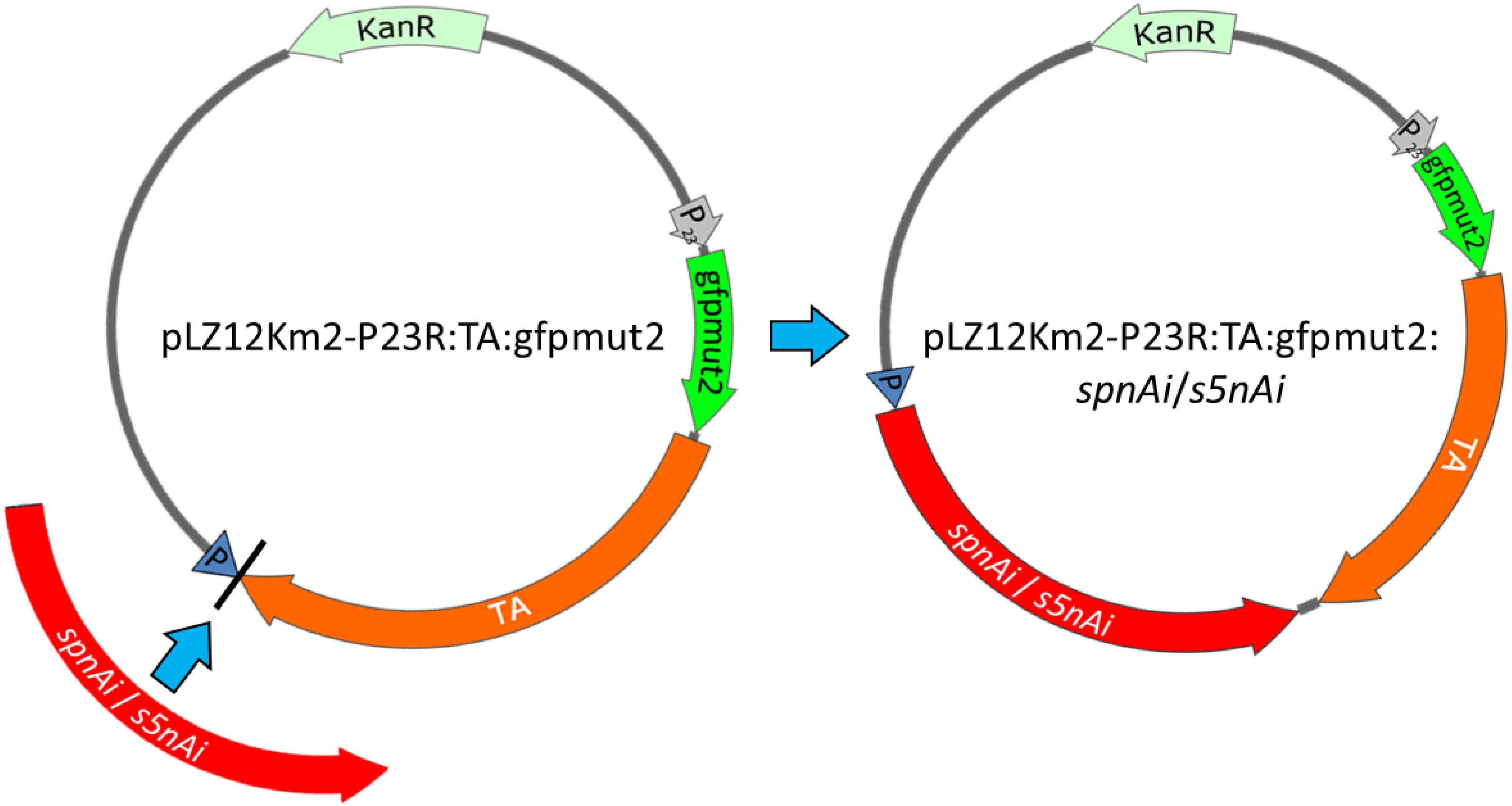

Figure S2. Plasmid construction for the generation of green fluorescent *S. iniae spnAi* and *s5nAi* complementation strains. Full length *spnAi* or *s5nAi* genes amplified from the WT *S. iniae* genome were cloned into pLZ12Km2-P23R:TA:gfpmut2 downstream of the original streptococcal promoter sequence. The constructed reporter plasmid was introduced into Δ*spnAi* or Δ*s5nAi* to generate green fluorescent *spnAi* and *s5nAi* complementation strain (*S. iniae* Δ*spnAi*:*spnAi* and *S. iniae* Δ*s5nAi*:*s5nAi*). KanR, kanamycin resistance gene; P23, strong lactococcal promoter; gfpmut2, a mutated version of the original green fluorescent gene; TA, toxin-antitoxin system; P, original streptococcal promoter of pLZ12Km2 plasmid.

Figure S3.

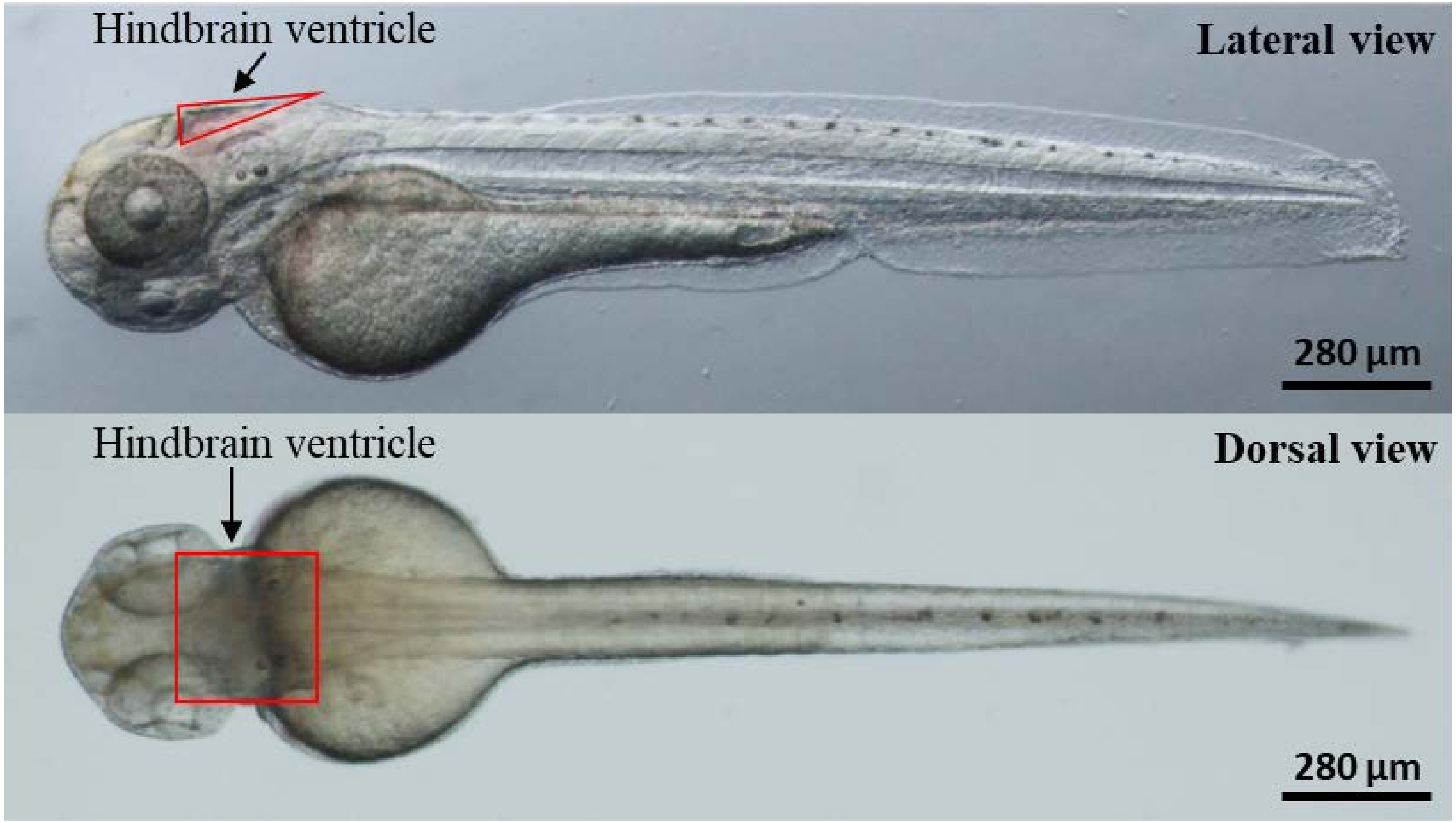

Figure S3. Lateral and dorsal view of a 2 dpf zebrafish larva. Confocal imaging of the area surrounded by the red square in dorsal view was performed in this study. Black arrow indicates the site of infection at hindbrain ventricle. Scale bar, 280 µm.

Figure S4.

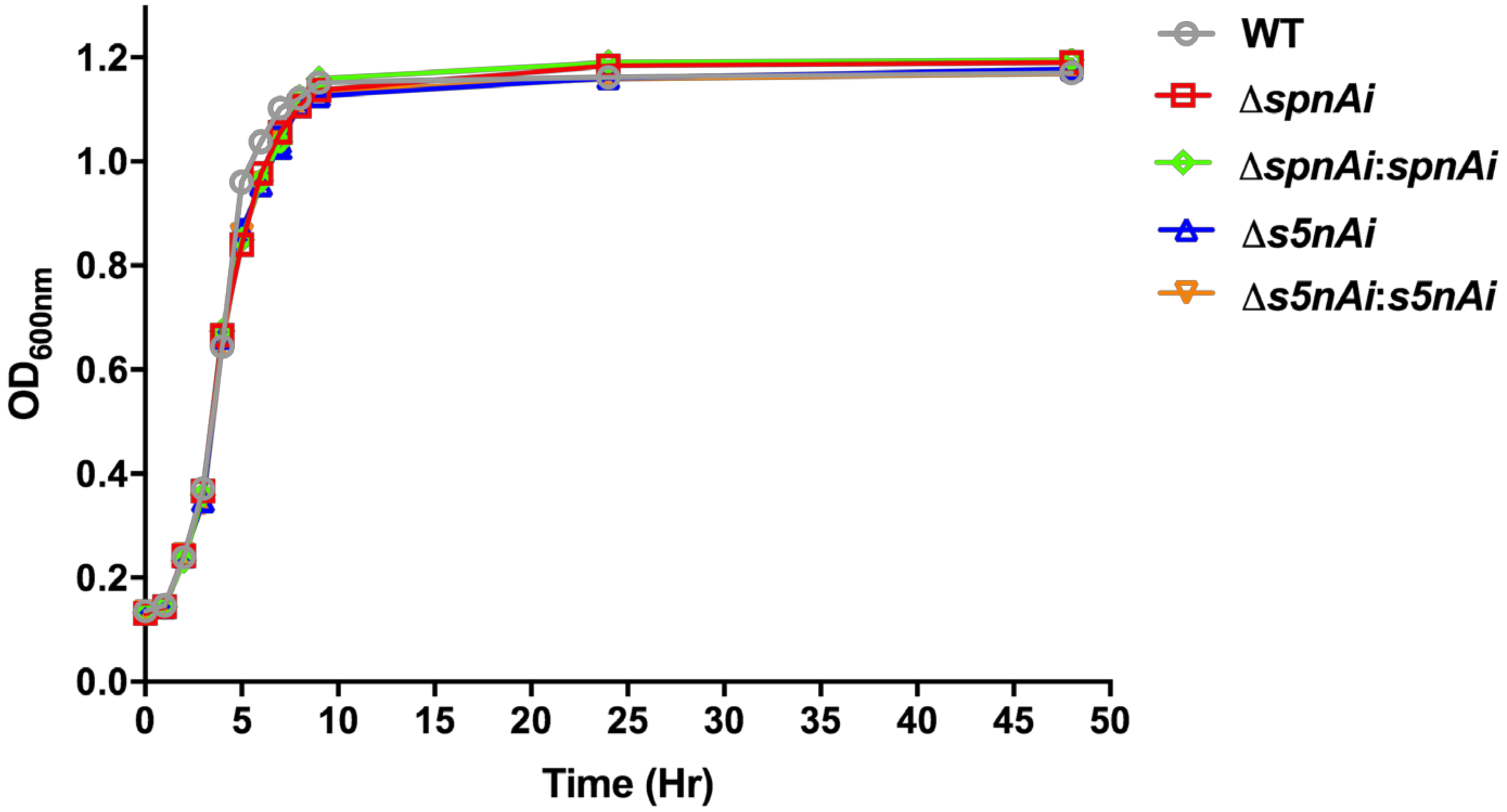

Figure S4. Growth rates of *S. iniae* strains. WT, Δ*spnAi*, Δ*s5nAi* and complementation strains were grown in THY+P medium. A sample was taken at the indicated time-points and the OD_{600nm} were measured. No significant difference was observed between the growth rate of the modified strains and the parental strain.

Movie S1.

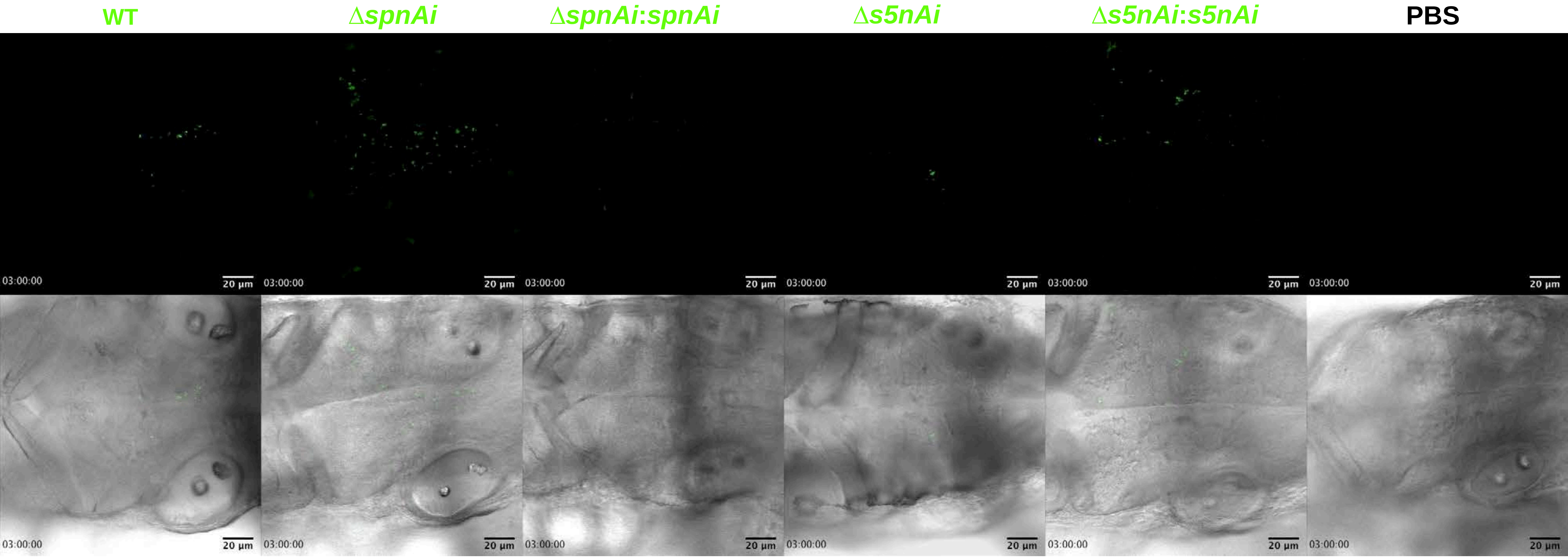

Movie S1. Time-lapse confocal imaging of 2 dpf zebrafish larvae infected with stably green fluorescent labelled *S. iniae* or mutant strains. The number in the lower-left corner of each frame indicates the time in hpi. Scale bar, 20 µm.
